# A cross-sectional observational study for ethno-geographical disparities in sleep quality, brain morphometry and cognition (a SOLACE study) in Indians residing in India, and South Asians and Europeans residing in the UK – a study protocol

**DOI:** 10.3389/fnagi.2024.1294681

**Published:** 2024-02-21

**Authors:** Rishabh Soni, Caroline Dale, Victoria Garfield, Nasreen Akhtar

**Affiliations:** ^1^Baldev Singh Sleep Electrophysiology Laboratory, Department of Physiology, All India Institute of Medical Sciences, New Delhi, India; ^2^Department of Non-Communicable Disease Epidemiology, London School of Hygiene and Tropical Medicine, London, United Kingdom; ^3^MRC Unit for Lifelong Health and Ageing, Institute of Cardiovascular Science, University College London, London, United Kingdom

**Keywords:** sleep, MRI, brain, cognition, sleep quality, SABRE, brain morphometry

## Abstract

**Introduction:**

As individuals age, their sleep patterns change, and sleep disturbances can increase the risk of dementia. Poor sleep quality can be a risk factor for mild cognitive impairment (MCI) and dementia. Epidemiological studies show a connection between sleep quality and cognitive changes, with brain imaging revealing grey matter volume reduction and amyloid beta accumulation in Alzheimer’s disease. However, most research has focused on Europeans, with little attention to other ethnic groups.

**Methods:**

This is a cross sectional study comparing effects across countries and ethnicities. Group 1 (*n* = 193) will be Indians residing in India (new participant recruitment), Group 2 will be South Asians residing in UK and group 3 will be Europeans residing in the UK. For group 2 and 3 (*n* = 193), data already collected by UK-based Southall and Brent REvisited (SABRE) tri-ethnic study will be used. For group 1, Pittsburgh Sleep Quality Index questionnaire (PSQI) will be used for assessment of sleep quality, Indian Council of Medical Research (Neurocognitive ToolBox) (ICMR-NCTB) for cognition testing and a 3 T MRI cerebral scan for brain morphometry. The data will be compared to sleep, cognitive function and brain MRI parameters from SABRE.

**Discussion:**

Racial and ethnic differences can impact the relationships of cognitive function, sleep quality and brain structure in older adults. Earlier studies have highlighted higher prevalence of poor sleep among black individuals compared to white individuals. Genetic or epigenetic mechanisms may contribute to these variations. Socio-cultural and environmental factors, such as neighbourhood, migration, lifestyle, stress and perceived discrimination may influence sleep patterns. The aim of the study is to examine the ethnogeographic variations in sleep quality, cognitive performance and brain morphometry among Indians living in India, and South Asians and Europeans residing in the UK.

## Introduction

Sleep disturbances may lower the threshold for dementia (Yaffe et al., 2014). Sleep duration pertains to the total hours spent asleep, whereas sleep quality is the subjective sense of feeling refreshed upon waking. Different manifestations of insomnia, including difficulties in falling asleep, maintaining sleep, or experiencing early morning awakenings, along with habitual snoring and other breathing issues during sleep, can lead to fragmented sleep patterns characterized by recurrent brief awakenings. These factors contribute to poor-quality sleep. The quality of sleep of an individual can serve as a risk factor for the development of mild cognitive impairment (MCI) and dementia (Scullin and Bliwise, 2015; Tsapanou et al., 2020; Simmonds et al., 2023). Difficulty in initiation of sleep is related to impaired global cognition (Nebes et al., 2009; Chang-Quan et al., 2012; Auyeung et al., 2013; Blackwell et al., 2014), worse working memory executive functions (Schmutte et al., 2007; Gamaldo et al., 2010; Rana et al., 2018), and verbal fluency. Early morning awakening has been found to be associated with poor performance on executive function tests (Ling et al., 2016), and with cognitive impairment in mid and late life (Jelicic et al., 2002). Excessive daytime sleepiness is also correlated with poor executive function and memory-based tasks in older adults (age 60 and above) (Ohayon, 2002; Okamura et al., 2016).

Although the amount of sleep varies individually, gender has been one of the factors underlying the variation. Some studies have reported that women tend to have longer sleep latency (Silva et al., 2008) and total sleep time (Bixler et al., 2009) as compared to men. Prevalence of insomnia and sleep disturbances is reported to be higher in women as compared to men (Ohayon, 2002; Madrid-Valero et al., 2017; Tang et al., 2017; Zeng et al., 2020).

Epidemiological studies have revealed a significant correlation between sleep quality and brain structure, as visualized by magnetic resonance imaging (MRI) (Tsapanou et al., 2020). Longer sleep latency and midnight awakenings are associated with low grey matter volume in insula and significant Aβ (amyloid beta) accumulation in prefrontal areas which are linked to Alzheimer’s disease (AD), the most common form of dementia (Branger et al., 2016; Rigat et al., 2023). Recent studies on older cohorts have revealed an association between daytime sleepiness and reduced grey matter and cortical volume (Tsapanou et al., 2020). In one study, difficulty in falling asleep was associated with cognitive impairment, and was more pronounced in men (de Souza Medeiros et al., 2022). Correlation of insomnia and atrophy of cortical and subcortical grey matter has also been documented (Grau-Rivera et al., 2020).

A small proportion of studies have reported ethnic differences in these parameters. A previous study using Southall and Brent REvisited (SABRE) tri-ethnic data has reported that Indian Asians, as compared to Europeans, have higher risk of mortality if reporting difficulty falling asleep and snoring problems (Garfield et al., 2019). Another study found that the quality of sleep, specifically snoring during middle age, is linked to the onset of type 2 diabetes in later life, even when accounting for established risk factors for type 2 diabetes. This correlation is evident in South-Asians but is not observed among Europeans and African-Caribbeans (Ong et al., 2021). As the incidence of dementia is increasing worldwide, it is important to understand the geographic, racial and cultural differences in sleep quality, brain health, and cognition.

The majority of the studies investigating the association of sleep quality, brain structure and cognitive function have been conducted in Europeans, and little attention has been paid to other ethnic groups. In Asian population, in a cross-sectional, community-based study in older Japanese adults, excessive daytime sleepiness was been found to be associated with subjective memory impairment (Okamura et al., 2016). Few, if any, studies to date have been conducted in the Indian Asian population with similar parameters. It is thus essential to acquire fresh data from the Indian population residing in India. The purpose of this study is to assess the variations in the relationship between sleep quality, cognitive performance, and brain structure among three specific population groups: Indians residing in India, South Asians residing in the UK, and the white European population residing in the UK.

### Ethics registration

IEC-66/14.01.2022 (AIIMS India) and 14/LO/0108 (UCL UK).

## Methods and analysis

### Study design

**Figure 1:**
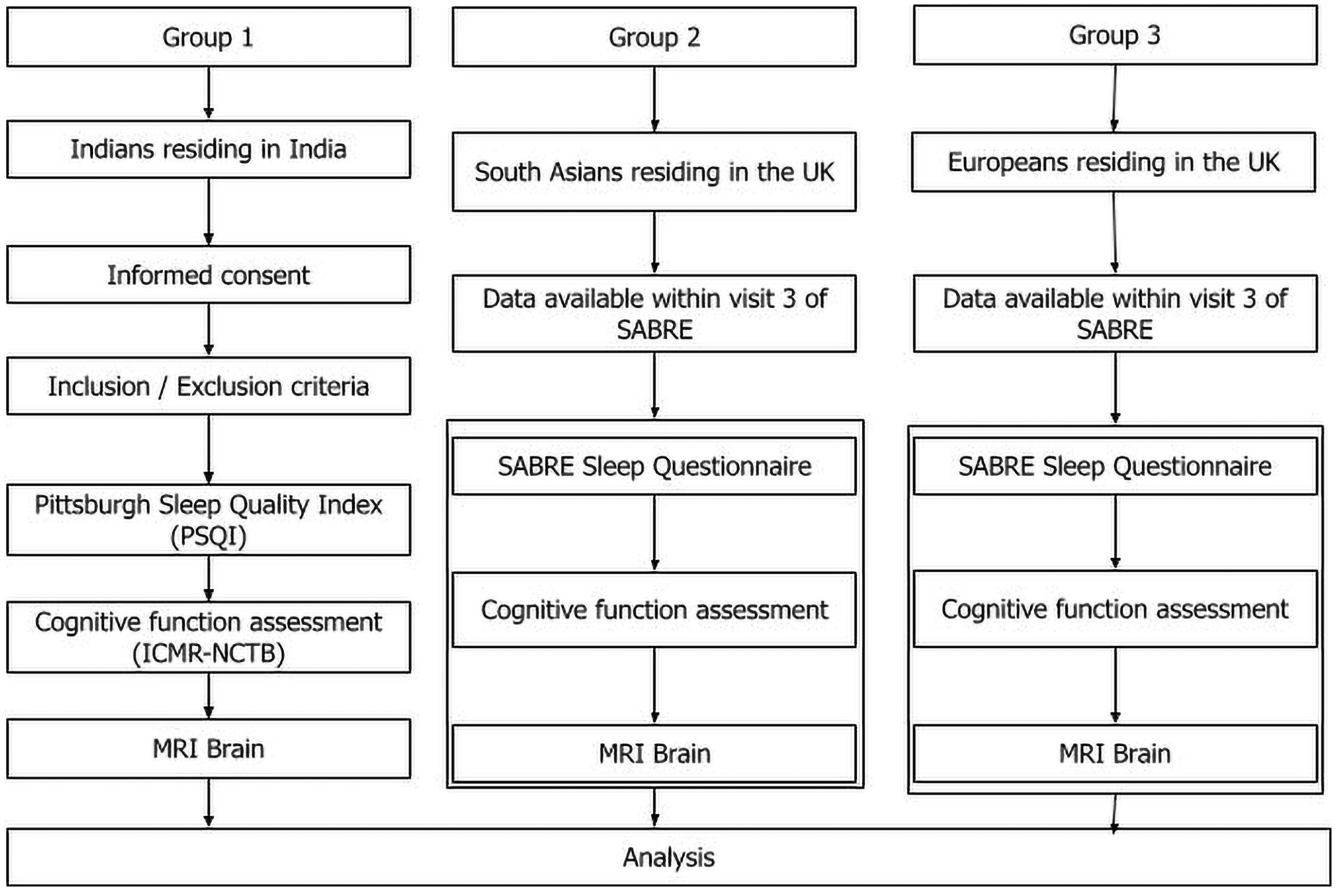
Flow diagram of the study. The study design has three arms: Indians residing in India, South Asians residing in the UK, and Europeans residing in the UK. The data for South Asians residing in UK and European population is already available in the SABRE BioBank. New data on Indian population in India will be collected at AIIMS after informed consent. Sleep quality, cognitive function, and brain MRI of all three groups will be compared.

This is a cross sectional study comparing effects across countries and ethnicities. Three groups will be compared in this study. Group 1 will be Indians residing in India (New participant recruitment), Group 2 will be South Asians residing in UK and group 3 will be Europeans residing in the UK. For group 2 and 3, data already collected by Southall and Brent REvisited (SABRE) tri-ethnic study will be used. Figure 1 displays a flowchart depicting the study design.

### Setting

New data of Indians residing in India will be collected afresh by new participant recruitment at a tertiary care center in India. Data acquisition will begin after obtaining the ethical approval from the Institute Ethics Committee. Data from the UK-based Southall and Brent REvisited (SABRE) tri-ethnic study at visit 3 (2014–18) will be analyzed in parallel with the new data collected in India.

### Selection of participants

#### Group 1

New participants in India will be recruited from out-patient departments of neurology, geriatric medicine memory clinic, healthy relatives of patients in the clinics at a tertiary care center, community outreach services, and, via advertisements and social media invitations. Participants will be screened for inclusion and exclusion criteria by history and physical examination. Inclusion criteria will be individuals of both sexes aged between 50 and 80 years, matching the age group of participants in the SABRE study. Exclusion criteria will be non-ambulatory patients, individuals diagnosed with major depression or general anxiety disorder, family history consistent with autosomal dominant Alzheimer’s Disease (AD), a history of any psychiatric condition, individuals currently using psychotropic medication and presence of MRI incompatible implants. Written informed consent will be obtained from all participants.

#### Group 2 and 3

Will consist of South Asians (Group 2) and Europeans residing in the UK (Group 3) at the 20-year follow-up in the Southall and Brent Revisited (SABRE) study. This study was first conducted between 2008 and 2012. It is a multiethnic community-based prospective cohort comprising older Europeans, South-Asians, and African-Caribbeans from London. SABRE begun in the late 1980s and then they were followed up twice, once at 20 years (this data will not be used in the current study) and then again between 2014-2018 and these are the data which will be analyzed for the study. The primary objective of this study is to investigate ethnic differences in cardiometabolic disorders. The median follow-up period for participants for the current study is 19 years, with an interquartile range (IQR) of 15 to 20 years. Participants’ ethnicity was initially determined by interviewers based on grandparental origin and subsequently confirmed by the participants themselves. Among the South-Asian participants, the majority are Punjabi Sikhs (52%), followed by Gujarati or Punjabi Hindus (20%), Muslims (15%), and other South-Asians (15%). The survivors at the follow-up assessment range from 57 to 90 years old. A comprehensive cohort profile and additional follow-up details are found in previous publications (Tillin et al., 2012, 2013).

## Measures

### Exposure: sleep quality

#### Group 1

Sleep quality will be assessed in the new participants in India using Pittsburg Sleep quality questionnaire. It is a subjective sleep quality assessment scale which includes 19 items that assess perceived sleep disturbance over the preceding 4 weeks. Seven component scores (i.e., sleep quality, sleep latency, sleep duration, habitual sleep efficiency, sleep disturbances, use of sleep medication, daytime dysfunction) are summed to derive a PSQI global score of subjective sleep quality (range 0–21 points). A global score of less than or equal to five is an indicator of good quality sleep. A weighted sleep quality score will be created.

#### Group 2 and 3

Within the SABRE cohort, participants responded to four questions regarding sleep quality during the baseline assessment. These questions assessed whether individuals experienced difficulty falling asleep, woke up too early, felt tired upon waking, and snored within the past 30 days. The first three questions were adapted from Jenkin’s Sleep Questionnaire (Jenkins et al., 1988), a concise, validated, reliable, and commonly used tool for evaluating sleep disturbances. A weighted sleep quality score will be created.

### Outcome

There are two outcomes- cognitive function and brain morphometry.

### Cognitive function

#### Group 1

The Indian Council of Medical Research-National Cognitive Test Battery (ICMR-NCTB) incorporates tests of the major cognitive domains of attention, executive functions, memory, language, and visuospatial skills adapted and translated into five Indian languages: Hindi, Bengali, Telugu, Kannada, and Malayalam. The ICMR-NCTB consists of a range of tests that evaluate the major cognitive domains: (a) tests of cognition for the various domains of attention-executive functions: Trail Making Test A & B (TMT A & B) and Category Fluency; memory: Verbal Learning Test-Total Learning and Delayed Recall (VLT-TL & DR) and Modified Taylor Complex Figure Test-Delayed Recall (MTCF-DR); and language (Picture Naming Test-PNT) and visuospatial skills (Modified Taylor Complex Figure test); and (b) questionnaires on behavior and functional activities: Geriatric Depression Scale (GDS), Instrumental Activities of Daily Living-Elderly (IADLE), Neuropsychiatric Inventory (NPI), Informant Questionnaire on Cognitive Decline in Elderly (IQCODE), and RAND Short Form Health Survey (RAND SF-36). This test battery was developed to screen and diagnose dementia and mild cognitive impairment in the early stages, across the country in India, and to be suitable for conducting global collaborative research in cognitive disorders (Verma et al., 2021).

#### Group 2 and 3

Cognitive function as been assessed in the SABRE cohort with a test battery that has been previously validated for cross cultural settings (Stewart et al., 2001). The following domains have been tested: global/overall function (Community Screening Instrument for Dementia [CSID] cognitive assessment); verbal memory (immediate and delayed verbal recall [CERAD 10-word]), visual memory (picture recognition); fluency (animal naming), processing speed (color trail-making A and B); and working memory (forward and backward digit span).

### Brain morphometry using magnetic resonance imaging

#### Group 1

All participants will undergo an MRI scan using a 3 T scanner (Philips, Achieva). MR protocol for T1 weighted images using 32 channel head coil will consist of a slice thickness of 1 mm, echo time (TE) =3.7 ms, repetition time (TR) =8.1 ms, flip angle = 8°, voxel size = 1 × 1 × 1 mm^3^ (acquired); 1 × 1 × 1 mm^3^ (reconstructed) and field-of-view (FOV) =240 × 240 × 180 mm^3^. MR protocol for T2 weighted images will consist of TE = 257 ms, TR = 2,500 ms, voxel size = 1 × 1 × 1 mm^3^ (reconstructed) and FOV = 250 × 250 × 175 mm^3^. Any images with motion artifacts will be excluded. Computational Anatomy Toolbox (CAT-12) will be used for cortical and subcortical analysis of T1 weighted images. This toolbox covers diverse morphometric methods such as voxel-based morphometry (VBM), surface-based morphometry (SBM), deformation-based morphometry (DBM), and region- or label-based morphometry (RBM). This will provide the parameters reported in the UK SABRE cohort: hippocampal volume, total brain volume, total intracranial volume, total grey matter volume.

#### Group 2 and 3

In the SABRE cohort, cerebral MRI has been performed based on the Cardiovascular Health Study protocol (Bryan et al., 1994). Parameters of interest are the total brain volume, total intracranial volume, total grey matter volume and total hippocampal volume. The MRI includes sagittal T1-weighted and axial T1-weighted, proton density, and T2-weighted images of 5-mm thickness with no gaps. For volumetric measures, 3-mm axial fluid-attenuated inversion recovery and coronal 1.5-mm 3-dimensional T1-weighted gradient echo images have been obtained. A third of the scans have been performed on a General Electric Signa HDxt 1.5 T scanner and the rest on a General Electric Discovery MR750 3 T scanner (GE Healthcare, Waukesha, WI). An automated segmentation protocol has been used to quantify total brain and hippocampal volume using FIRST in FSL 4.1 (Patenaude et al., 2011). Total brain volume (TBV) has been computed as the volume after skull stripping of the T1-weighted image using BET (Smith, 2002), in FSL 5.0. Table 1 shows the dependent variables.

**Table 1:** Dependent variables.

Neuroimaging (MRI)	Cognition (ICMR-NCTB)
Total intracranial volume Total brain volume Hippocampus volume Global gray matter volume	*Group 1*
	Montreal Cognitive Assessment
	Trail Making Test Black and White (A & B)
	Category Fluency Test
	Phonemic Fluency Test
	Verbal Learning Test
	TNI-93
	Picture Naming Test (PNT)
	Frenchay Aphasia Screening Test (FAST)
	Modified Taylor Complex Figure Test (MTCF)
	Line Bisection Test
	*Group 2 and 3*
	Community Screening Instrument for Dementia
	[CSID] cognitive assessment
	Verbal memory (immediate and delayed verbal recall)
	[CERAD 10-word]
	Picture recognition
	Animal naming
	Color trail-making A and B
	Forward and backward digit span

### Covariates

These will include demographic factors (age, sex, socioeconomic status), health behaviors (alcohol consumption and smoking) and comorbidities (cardiovascular disease, type-2 diabetes, hypertension, hyperlipidaemia, body mass index). Weight, height, blood pressure, abdominal circumference, neck circumference, waist:hip ratio, and medications will be recorded before the conduct of study in group 1. Records of medications will be maintained to investigate the effect of their medication on their sleep structure. For group 2 and 3, these variables have been obtained at the third visit (2014–18). Table 2 shows the sociodemographic variables for group 1 (age, smoking, alcohol consumption, living circumstances, education, household income, and socioeconomic status).

**Table 2:** Sociodemographic variables.

Variable	Values
Age	Years
Identified gender	Female/Male/Prefer not to say
Smoking	Current/ever
Alcohol consumption	Current (volume per day)/binge drinking/ever
Living circumstances	Alone / with spouse / with family
Education	Years of education, Highest qualifications
Household Income	Indian Rupees (INR)
Socioeconomic Status	Upper (I), Upper Middle (II), Lower Middle (III), Upper Lower (IV), Lower (V)
Years of education	Years
Ongoing medical issues (such as CVD, diabetes, hypertension, hyperlipidaemia)	Name of the issue (to be taken as controlled variable)
Medications	Name of the drug
Weight	Kilograms
Height	Centimeters
Blood pressure	mmHg
Abdominal circumference	Centimeters
Neck circumference	Centimeters
Waist:hip ratio	

### Sample size

As the comparison of the study parameters between Indians in the UK and Indians in India is a novel proposition, there is no meta-analysis available for this kind of comparison. We would like the acquisition of new data on the Indian side to approximate the size of the sample already in the SABRE study (population with MRI also), that is 193. Thus, we would like to keep the sample size at 193. SABRE currently possesses the data of 193 Indian Asians and 287 Europeans with all the required measurements.

### Statistical methods

Exposures will be sleep quality questions from Pittsburgh Sleep Quality Index (PSQI). We will create a weighted sleep quality score as per the analysis in the SABRE Cohort (Ong et al., 2021; Topriceanu et al., 2021). The outcomes will be the following: (a) neuroimaging/morphological outcomes: hippocampal volume, total brain volume, total intracranial volume, total grey matter volume; and other parameters (b) cognitive function: verbal memory, language, reasoning and delayed visual recall, attention, global function (to screen for dementia) and functional literacy. Confounding factors will include demographic factors (age, sex, socioeconomic status, education) and health behaviors (alcohol consumption, smoking) and comorbidities (cardiovascular disease, type-2 diabetes, hypertension, hyperlipidemia, body mass index). To avoid bias in the study, we will use gender as a covariate in one of the sub analyses.

We will fit three sequential models. Model 1 will include adjustment for demographic factors, model 2 will additionally include smoking + alcohol, model 3 will additionally include co-morbidities. We will use linear regression to examine the correlation of sleep quality with neuroimaging outcomes. All three models will be stratified to ethnic groups; hence the SABRE analysis will be done independently in Europeans and Indian Asians. The results which will be obtained from linear regression models for each ethnic group will include standardized beta coefficients with confidence intervals of 95%.

To account for multiple testing within each outcome group (cognition and MRI), we will apply Bonferroni corrections. With seven cognitive outcomes and four MRI outcomes, the adjusted alpha levels will be 0.05/7 = 0.007 for cognition and 0.05/4 = 0.013 for MRI. This helps maintain an overall alpha level of 0.05 while controlling for family-wise error.

For ethnic comparisons, we will perform Cochran’s Q heterogeneity tests. This statistical test will assess whether the effect sizes (represented by standardized beta coefficients in our linear regression models) significantly differ between Indians in India, Indian Asians in the UK, and Europeans in the UK.

## Discussion

The aim of the study is to examine the ethnogeographic variations in sleep quality, cognitive performance and brain morphometry among Indians living in India, and South Asians and Europeans residing in the UK. We will assess how sleep quality affects cognitive function and brain structure in these populations.

The mechanisms linking sleep and brain structure and cognition encompass a range of crucial processes. During sleep, synaptic pruning and plasticity refine neural connections (Paolicelli et al., 2011), while hormone release, particularly growth hormone and cortisol, influences brain development and maintenance (Kim et al., 2015). In young adults, poor sleep quality may be associated with a significant risk of developing obstructive sleep apnoea (Aggarwal et al., 2021) and frequent arousals (Bhat et al., 2022). Sleep supports neuroplasticity, reorganizing neural networks, and promoting gray matter changes. The hippocampus is closely tied to sleep, aiding memory consolidation, while sleep facilitates the clearance of toxic substances like beta-amyloid (Aggarwal et al., 2021). Proper sleep maintains optimal brain connectivity, supports myelin sheath integrity, and may even promote neurogenesis (Payne and Nadel, 2004; de Vivo and Bellesi, 2019).

Impaired sleep quality has been shown to impact cognitive performance differently in men and women. Some studies suggest that women may be more resilient to the cognitive effects of sleep quality than men (Alhola and Polo-Kantola, 2007; Bixler et al., 2009), whereas others suggest the opposite (Ferrara et al., 2015; Hajali et al., 2019). These studies indicate that gender may impact sleep architecture, and have consequences on cognitive performance. Research on sleep differences between genders holds profound significance for public health, as sleep-related issues are associated with a range of conditions, including cognitive decline, mood disorders, and metabolic disturbances. Gender differences will be analyzed in our study.

The mechanism behind the ethnic differences in sleep quality, brain morphometry and cognition is still under investigation. One review proposed a conceptual model positing that social and environmental factors, including neighborhood characteristics, occupational stress, treatment accessibility, and perceived discrimination, may exert influence over sleep quality, duration, and the prevalence of sleep disorders (Kingsbury et al., 2013; Siow et al., 2022). However, additional long-term studies are necessary to confirm and strengthen the validity of these findings. Other studies have hypothesized that cultural and environmental factors (such as lifestyle, diet, stress, and exposure to different light and noise environments) may influence the relationship between sleep, cognitive performance, and brain morphometry in these distinct populations (Gildner et al., 2014). For Indians residing in the UK, it has been suggested that the differences might be due to the effects of migration and acculturation. This could involve assessing how the adoption of a new cultural and environmental context impacts these factors. Racial and ethnic variations in sleep quality and sleep pattern may affect cognitive function differentially. Inadequate sleep quality may contribute to impaired memory among older adults. Poor sleep tends to be more prevalent among black individuals compared to white individuals, potentially due to variations in health and psychosocial factors (Hokett and Duarte, 2019; Hokett et al., 2022). Moreover, black participants exhibited higher within-person variability in their sleep quality compared to white participants. This increased variability in sleep quality was strongly associated with decreased neural activity related to memory in black participants. The study further revealed that greater variability in sleep quality was linked to poorer memory performance, particularly in older adults. Genetic or epigenetic mechanisms have been reported to contribute to variations in sleep quality and their effects on cognitive function and brain structure in different ethnicities (Zhang et al., 2023). This study will look into some factors that may be contributing to the racial and ethnic differences such as socio-economic status, literacy, sleep quality, etc.

Studying ethnic and racial differences in the relationship between sleep, cognition, and brain structure is important for several reasons. Understanding these disparities can lead to more equitable healthcare and interventions. Insights from this research can inform public health policies (Akhtar and Mallick, 2019) and interventions aimed at improving sleep health and cognitive outcomes for different communities, ultimately enhancing overall well-being. The cross-sectional, cross-country study we are proposing may also help prospectively in the establishment of a cohort in India on the lines of the SABRE cohort in the UK. The cohort can then be used to study the cause-and-effect relationships between current dependent (outcome) and independent (exposure) variables. If a correlation between poor sleep quality and impaired cognition/brain morphometry is found, subsequent studies can target sleep quality as a modifiable risk factor to reduce cognitive decline. Sleep is essential for cognitive function, and impaired sleep quality can lead to a variety of cognitive deficits, including impaired attention, memory, and decision-making (Deak and Stickgold, 2010; Pérez-Carbonell and Iranzo, 2023). Our study might be helpful to determine how disturbed sleep disrupts the normal process of memory consolidation and how sleep quality is correlated with brain function. Our study will be one of the first to examine the association of sleep quality, brain structure, and cognition in a sample of healthy adults from India, a country with a large and diverse population and a high burden of cognitive impairment and dementia. This study will explore the crucial impact of cultural and geographical contexts on sleep patterns, cognitive decline, and brain structure. By assessing differences across ethnicities, races, and nations, it will shed light on how these vital parameters are impacted by diverse societal and environmental factors. This goes beyond identifying sleep quality as simply a correlate of cognitive decline and dementia; it will offer valuable insights into the interplay between these elements in distinct populations. This knowledge will pave the way for developing culturally sensitive interventions that optimize sleep quality, enhance cognitive health, and improve overall well-being in older adults around the world. Ultimately, this study will empower us to move beyond one-size-fits-all healthcare approaches and embrace personalized strategies tailored to the specific needs of each individual within their unique cultural and geographic context.

## Ethics statement

The studies involving humans were approved by Institute Ethics Committee of Postgraduate Research (IECPG), AIIMS, New Delhi. The studies were conducted in accordance with the local legislation and institutional requirements. The participants provided their written informed consent to participate in this study.

## Author contributions

RS: Writing – original draft, Writing – review & editing. CD: Writing – review & editing. VG: Conceptualization, Funding acquisition, Supervision, Writing – original draft, Writing – review & editing. NA: Funding acquisition, Methodology, Supervision, Writing – original draft, Writing – review & editing.
